# The search for early markers of AD: hippocampal atrophy and memory deficits

**DOI:** 10.1017/S1041610214000623

**Published:** 2014-07

**Authors:** Josephine Barnes, Nick C. Fox

**Affiliations:** Dementia Research Centre, Department of Neurodegenerative Disease, UCL Institute of Neurology, Queen Square, London WC1N 3BGUnited Kingdom Email: n.fox@ucl.ac.uk

## Abstract

There is increasing interest in finding markers of Alzheimer's disease (AD) that are discriminative even at an early, pre-dementia stage. This interest is driven partly by a desire to improve clinical diagnosis in more mildly affected individuals, and also by the recent paradigm shift in thinking about clinical trials for AD. This shift is a result of concern that the recent failures of high-profile clinical trials conducted in patients with mild to moderate AD may have been because therapy was “too little, too late.” The implication being that if only treatments had been trialled earlier they would have had a greater chance of success. Certainly, lessons from other aspects of medicine have shown that treatments may be most, or in some cases only, effective if given early in disease. If we did have therapies that could slow disease progression at a very early stage that would increase the interest in early markers of disease. Ideally, such therapies would be given when the minimum of functional decline and irreversible neuronal loss had already occurred. From economic and public health standpoints, delaying symptom onset would be very important: a delay of five years has been estimated to reduce projections for prevalence of symptomatic AD by about 50% (Brookmeyer *et al.*, 1998).

There is increasing interest in finding markers of Alzheimer's disease (AD) that are discriminative even at an early, pre-dementia stage. This interest is driven partly by a desire to improve clinical diagnosis in more mildly affected individuals, and also by the recent paradigm shift in thinking about clinical trials for AD. This shift is a result of concern that the recent failures of high-profile clinical trials conducted in patients with mild to moderate AD may have been because therapy was “too little, too late.” The implication being that if only treatments had been trialled earlier they would have had a greater chance of success. Certainly, lessons from other aspects of medicine have shown that treatments may be most, or in some cases only, effective if given early in disease. If we did have therapies that could slow disease progression at a very early stage that would increase the interest in early markers of disease. Ideally, such therapies would be given when the minimum of functional decline and irreversible neuronal loss had already occurred. From economic and public health standpoints, delaying symptom onset would be very important: a delay of five years has been estimated to reduce projections for prevalence of symptomatic AD by about 50% (Brookmeyer *et al.*, 1998).

We now know there is a long prodromal period to AD when pathology (amyloid plaques and neurofibrillary tangles) gradually and progressively accumulates in the brain. While the exact timing and sequencing of the different pathological changes that occur remains to be determined precisely, evidence from a variety of sources suggests that cerebral plaques and tangles appear many years before symptoms. Positron emission tomography (PET) imaging and cerebrospinal fluid (CSF) studies have provided *in vivo* evidence to back up earlier findings from autopsy studies and collectively these imply that pathological changes are present and detectable as early as 10–15 years before symptoms. Some years after these changes first appear, but still prior to symptoms, the downstream structural and functional consequences become apparent. In particular, longitudinal imaging studies have shown that pathologically increased hippocampal atrophy is detectable three to five years before diagnosis. These findings have led to a reframing of AD research criteria so that a diagnosis of AD no longer requires dementia to already be present. The new criteria state that if supportive biomarker or imaging changes are present then a diagnosis of prodromal AD can be made (Dubois *et al.*, 2010; Albert *et al.*, 2011). Hippocampal atrophy on structural imaging (MRI (magnetic resonance imaging) or CT (computed tomography)) is one of the supportive imaging features included in both these sets of research criteria.

In this month's issue of *International Psychogeriatrics*, Ferrarini and colleagues describe the results of a population-based study of hippocampal volume and shape abnormalities in elderly subjects with memory deficits (Ferrarini *et al*., 2014). Although a large number of studies have demonstrated that hippocampal atrophy is associated with AD and in those with memory deficits is predictive of future cognitive decline, these have largely been using highly selected clinical cohorts and community-based studies are fewer in number. This is important because the vast majority of those who are given a clinical diagnosis of AD are not seen at tertiary referral or AD research centers. Furthermore, the wider population will often have multiple co-morbidities which may mean they are excluded from selective studies. Ferrarini and colleagues sought to address this issue and additionally to assess and see changes in the shape of the hippocampus as well as hippocampal volume loss. The cohort used in the paper is relevant to the general population at risk of late-onset AD in many ways; it is multi-center (from 12 different rural and urban municipalities) and subjects were recruited from an epidemiological study rather than clinics and the age range was from 65 to 84 years. In this cross-sectional study, the authors compared three different groups. There were 75 “cognitively normal” subjects, 31 subjects who had memory deficits that were felt to be consistent with early AD, and 31 subjects who had memory deficits but either did not have a history suggestive of a gradual decline in memory or who had medical of psychiatric co-morbidities that presumably were considered as potential confounds.

So what did they find? Both the memory-impaired groups had lower hippocampal volumes than the control group by about 7% to 8% and, when considering all the subjects as one group, lower memory scores were correlated with lower hippocampal volume. This is indeed consistent with previous studies and suggests that these very mildly affected subjects do have pathological hippocampal losses. Hippocampal volumes discriminated individuals with memory deficits from healthy controls with an accuracy of about 0.6 to 0.7 (area under the receiver operating characteristic curve). The authors did not find that their measures of hippocampal shape were particularly useful in differentiating the different groups. This may be because, for methodological reasons, the shape measures were insensitive to focal losses or alternatively it may suggest that very early structural changes in AD are rather diffusely distributed across the hippocampus. A strength of this study is the fact that the subjects were drawn from a representative sample of the general elderly Italian population. So, as the authors state, the results provide support for the “validity of hippocampal volumetry as a biomarker of memory impairment in the general population”. What the study cannot yet tell us is what the value of hippocampal volumetry is in terms of early diagnosis of AD in this population. Importantly however, the project is ongoing and the authors report that clinical follow-up is being collected from all the subjects. Results from this follow-up will make these baseline data much more valuable. At the individual patient level, it is not whether or not a marker, be it an imaging measure such as hippocampal atrophy, or a CSF, or blood biomarker, is associated with memory deficits but rather whether or not the marker predicts future clinical decline and can guide management. Almost 30 years on since Ball and colleagues suggested that AD should be defined as a hippocampal dementia, we are still working out how best (and when) hippocampal measures can be used to improve early diagnosis (Ball *et al.*, 1985). This paper shows both the potential and some of the cautions that need to be associated with the use of hippocampal measures in very early disease.

The fact that we are entering a new era of therapeutic trials in presymptomatic subjects will add greater urgency to our search for effective markers of disease and disease progression at this early stage. These trials will incorporate the increasing range of markers available: molecular imaging and CSF fluid biomarkers as well as hippocampal atrophy. These markers are currently trying to find their appropriate place in clinical practice. If an effective disease modifying therapy is found, that practice is likely to be transformed.

## Conflict of interest

None.
